# Study preferences and exam outcomes in medical education: insights from renal physiology

**DOI:** 10.1186/s12909-024-05964-4

**Published:** 2024-09-06

**Authors:** Sofie Fagervoll Heltne, Sigrid Hovdenakk, Monika Kvernenes, Olav Tenstad

**Affiliations:** 1 Department of Biomedicine, University of Bergen, Jonas Lies vei 91, Bergen, N- 5009 Norway; 2 Center for Medical Education, Department of Clinical Medicine, University of Bergen, Bergen, Norway

**Keywords:** Renal physiology, Teaching methods, Medical school, Learning resources, Academic achievement, Medical students, Active learning

## Abstract

**Background:**

Efficient learning strategies and resource utilization are critical in medical education, especially for complex subjects like renal physiology. This is increasingly important given the rise in chronic renal diseases and the decline in nephrology fellowships. However, the correlations between study time, perceived utility of learning resources, and academic performance are not well-explored, which led to this study.

**Methods:**

A cross-sectional survey was conducted with second-year medical students at the University of Bergen, Norway, to assess their preferred learning resources and study time dedicated to renal physiology. Responses were correlated with end-of-term exam scores.

**Results:**

The study revealed no significant correlation between time spent studying and overall academic performance, highlighting the importance of study quality over quantity. Preferences for active learning resources, such as Team-Based Learning, interactive lessons and formative assignments, were positively correlated with better academic performance. A notable correlation was found between students’ valuation of teachers’ professional competence and their total academic scores. Conversely, perceived difficulty across the curriculum and reliance on self-found online resources in renal physiology correlated negatively with academic performance. ‘The Renal Pod’, a locally produced renal physiology podcast, was popular across grades. Interestingly, students who listened to all episodes once achieved higher exam scores compared to those who listened to only some episodes, reflecting a strategic approach to podcast use. Textbooks, while less popular, did not correlate with higher exam scores. Despite the specific focus on renal physiology, learning preferences are systematically correlated with broader academic outcomes, reflecting the interconnected nature of medical education.

**Conclusion:**

The study suggests that the quality and strategic approaches to learning significantly impact academic performance. Successful learners tend to be proactive, engaged, and strategic, valuing expert instruction and active participation. These findings support the integration of student-activating teaching methods and assignments that reward deep learning.

**Supplementary Information:**

The online version contains supplementary material available at 10.1186/s12909-024-05964-4.

## Introduction

Medical students are under pressure to acquire knowledge and skills in many fields during medical school, which means they need to prioritize their time and study efforts wisely. To study effectively, students must be able to choose among a variety of learning resources. Traditionally, students’ preferred mode of learning has been attributed to learning styles described as relatively stable personality traits [1]. However, recent studies indicate that students adapt their study approaches based on contextual factors, such as the perceived importance of the study topic, its difficulty, stress levels, assessment methods, and identified learning needs [2, 3]. Research on teaching preferences among students yields mixed results regarding their inclination toward active learning methods versus passive formats such as lecturing [4–7].

Notably, students do not always utilize learning resources that correlate with better exam performance. This discrepancy might stem from misaligned assessment methods or students’ challenges in accurately assessing their learning needs [4, 8–10]. Investigating the relationship between students’ perceptions of learning resource usefulness and exam performance is likely to offer valuable insights for educators and students in selecting the most effective learning tools.

Our study specifically targets the learning of renal physiology, a subject of increasing importance due to the rising prevalence of chronic renal diseases, which poses a significant socioeconomic challenge [11]. This concern is compounded by a notable decline in nephrology fellowship enrollments [12–14], suggesting a potential future strain on the nephrology workforce. The complexity of renal physiology, and nephrology as a specialty, contributes to this issue. A survey of internal medicine subspecialty fellows revealed that 31% of the respondents found nephrology to be the most challenging physiology course in medical school, and 24% would have considered nephrology had it been taught effectively [13]. A revamped renal physiology course, incorporating diverse learning resources, yielded positive student attitudes toward both renal physiology and nephrology [12].

It is plausible that students who engage in active learning and utilize multiple resources also achieve better academically. Incorporating a range of active learning resources into the curriculum could enhance educational quality and, in the long run, boost the healthcare system, including nephrology recruitment. To our knowledge, the relationship between academic success and preferred learning resources has not been explored in the context of renal physiology. Our study aimed to examine the association between students’ study time, their perceptions of learning resource efficacy, and their summative assessment outcomes to identify characteristics of successful learners.

## Methods

### Study context

The study was conducted at the Faculty of Medicine, University of Bergen, which offers a six-year medical program. In 2015, an integrated curriculum was implemented, emphasizing basic sciences during the first two years and progressively integrating clinical exposure. The program is structured into 12 instructional units, divided into spring and fall semesters, with each year consisting of two semesters. Additionally, there are elective periods from the 6th semester (3rd year), consisting of four weeks each January, that allow students to delve into specific topics of interest.

The program is divided into two study tracks: one based entirely in Bergen and another called ‘Vestlandslegen,’ where students spend the first three years in Bergen and the last three years in Stavanger. This structure allows for diverse clinical exposure in different healthcare settings. Clinical practice is a significant component of the program, starting early in the education. From the fourth year, students are placed in extended clinical rotations across various specialties, including psychiatry, internal medicine, surgery, obstetrics/gynecology, pediatrics, general practice, and community medicine. These rotations take place in multiple hospitals, including Haukeland University Hospital in Bergen, Stavanger University Hospital, Førde Central Hospital, and Haugesund Hospital. The program also offers opportunities for international clinical placements in Uganda and Thailand during the final years.

Our study focused on MED4 (the 4th semester), where students encounter a broad spectrum of medical sciences, including the renal and urinary systems; cardiovascular, respiratory, endocrine, digestive, and reproductive systems; as well as energy and thermoregulation, nutrition, microbiology, environmental medicine, and general practice. Environmental medicine and general practice are assessed on a continuous basis with a pass/fail evaluation, while the rest of the subjects are assessed through a 33-credit, six-hour summative exam, held at the end of the semester. This exam includes a mix of short-answer questions requiring reasoning and predominantly reasoning-based multiple-choice questions (MCQs). The grading scale for MED4 ranges from A to F, where A represents excellent performance, B is very good, C is good, D is satisfactory, E is sufficient, and F is fail.

Teaching methods in MED4 include traditional lectures, TBL sessions, practical lab courses, quizzes, and online teacher-moderated discussion forums. At least half of the teaching time is dedicated to active learning methods to enhance student engagement and understanding. All lab courses and dissections are mandatory and include compulsory assignments that must be passed. A new and unique feature of the MED4 curriculum is the introduction of formative assignments with feedback. These activities are also mandatory and designed to train students in presenting solutions to academic questions to an instructor who is not a specialist in the field. The instructors receive prior instruction from specialists and are provided with a written guide to ensure consistency in feedback. Each semester, one session is held early, in the middle, and towards the end of the semester. During these sessions, students receive two central assignments from specific subject areas that have recently been covered. Students have 90 min to prepare their responses, and the preparation can involve any resources, including artificial intelligence tools. After the preparation period, students present their solutions in pairs to a faculty member. Each pair is allotted 15 min to present, with each student presenting one of the assignments for a maximum of five minutes. Feedback is provided on whether the presentation meets, falls below, or exceeds expectations regarding clarity and accuracy for non-specialists. After these sessions, a discussion forum is created for further questions, monitored by the task owner, with participation encouraged from the entire cohort.

The renal physiology segment of MED4 encompasses an in-depth exploration of renal function, including water and electrolyte balance and acid-base homeostasis. Students are required to engage in 3 mandatory Team-Based Learning (TBL) sessions and a full-day practical lab course. Additionally, they have the option to attend 4 interactive lessons structured as 1.5-hour flipped classroom sessions. These interactive lessons require preparation in the form of reading textbook chapters, watching instructional videos, or listening to selected podcasts that succinctly cover the content traditionally delivered in lectures. These optional interactive lessons are popular, with about two-thirds of the cohort attending regularly. Complementary learning resources for this segment include suggested study group assignments, a FAQ with previously answered student questions, and ‘The Renal Pod’ podcast series. This multi-faceted approach aims to provide a comprehensive understanding of renal physiology and prepare students for clinical practice.

### Data collection and study design

A 13-question survey was developed using SurveyXact to collect data for this cross-sectional study (supplementary file). The survey was designed in accordance with established guidelines [15, 16] while being sensitive to the study context to optimize relevance for the students being surveyed. To ensure validity, we informally tested the questions with students and colleagues, and consulted with experts from the Center for Medical Education at the University of Bergen. Reliability was promoted through the use of clear and unambiguous wording to ensure consistent responses.

The survey comprised both closed- and open-ended questions, designed to identify factors students considered most important for learning renal physiology, assess the perceived difficulty of renal physiology relative to other MED4 subjects, and determine the time dedicated to studying these areas. Additionally, it evaluated the perceived utility of different learning resources, the time invested in preparing for learning activities in renal physiology, the effectiveness and optimal episode length of the renal podcast, and recommendations for the formative assignment pilot. The learning resources included in the survey were chosen based on their availability to students, previous usage in the course, and their relevance as identified in preliminary discussions with faculty and students.

The survey was distributed to 201 students enrolled in MED4. Participation was voluntary, and unique exam identifiers enabled the correlation of survey responses with exam outcomes. Anonymity was ensured, as these identifiers could not be traced back to individual students’ identities. Respondents were informed that their participation would not have any bearing on their grades, with no incentives provided. Out of the 201 students, 6 withdrew before the exam, leaving 195 students. Of these, 189 attended the exam and were considered eligible for analysis, resulting in a response rate of 38.1%.

### Data analysis

**Fig. 1:**
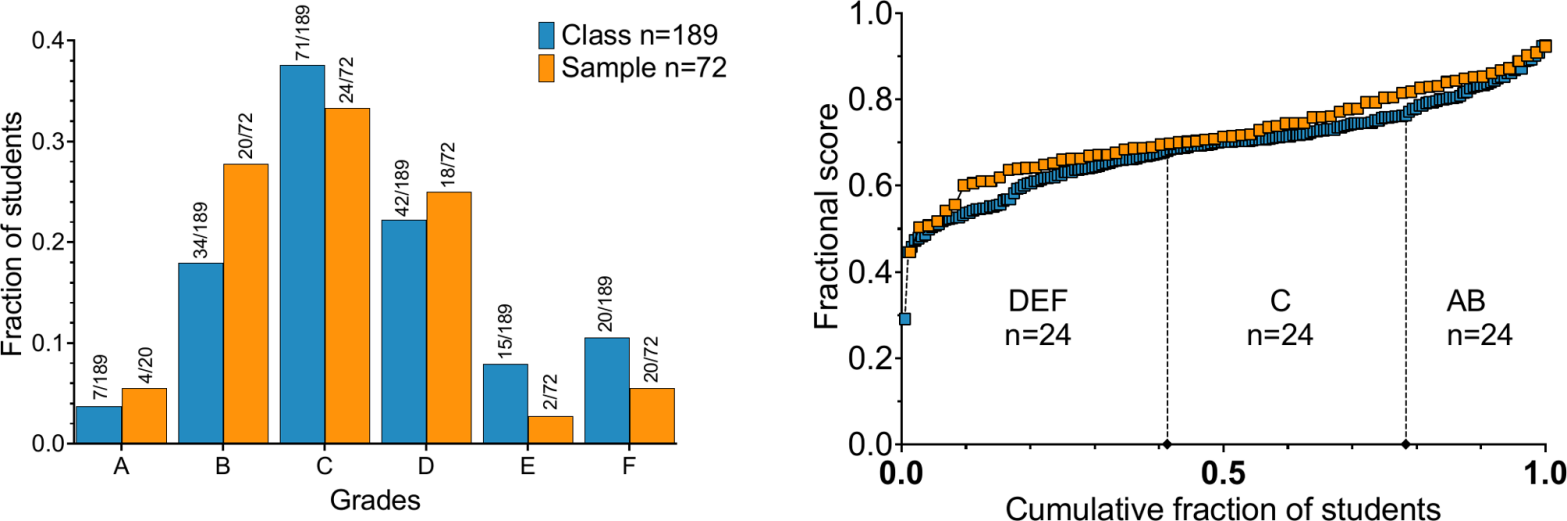
Comparison of exam grades and scores between the whole class and the sample population. **Left panel**: Grades are categorized into AB (*n* = 24/*n* = 41), C (*n* = 24/*n* = 71), and DEF (*n* = 24/*n* = 77). **Right panel**: Scores, represented as fractions of the total obtainable score, are plotted in ascending order against the cumulative fraction of students for the whole class (*n* = 189) and the sample population (*n* = 72), with values ranging from 1/n to n/n

**Fig. 2:**
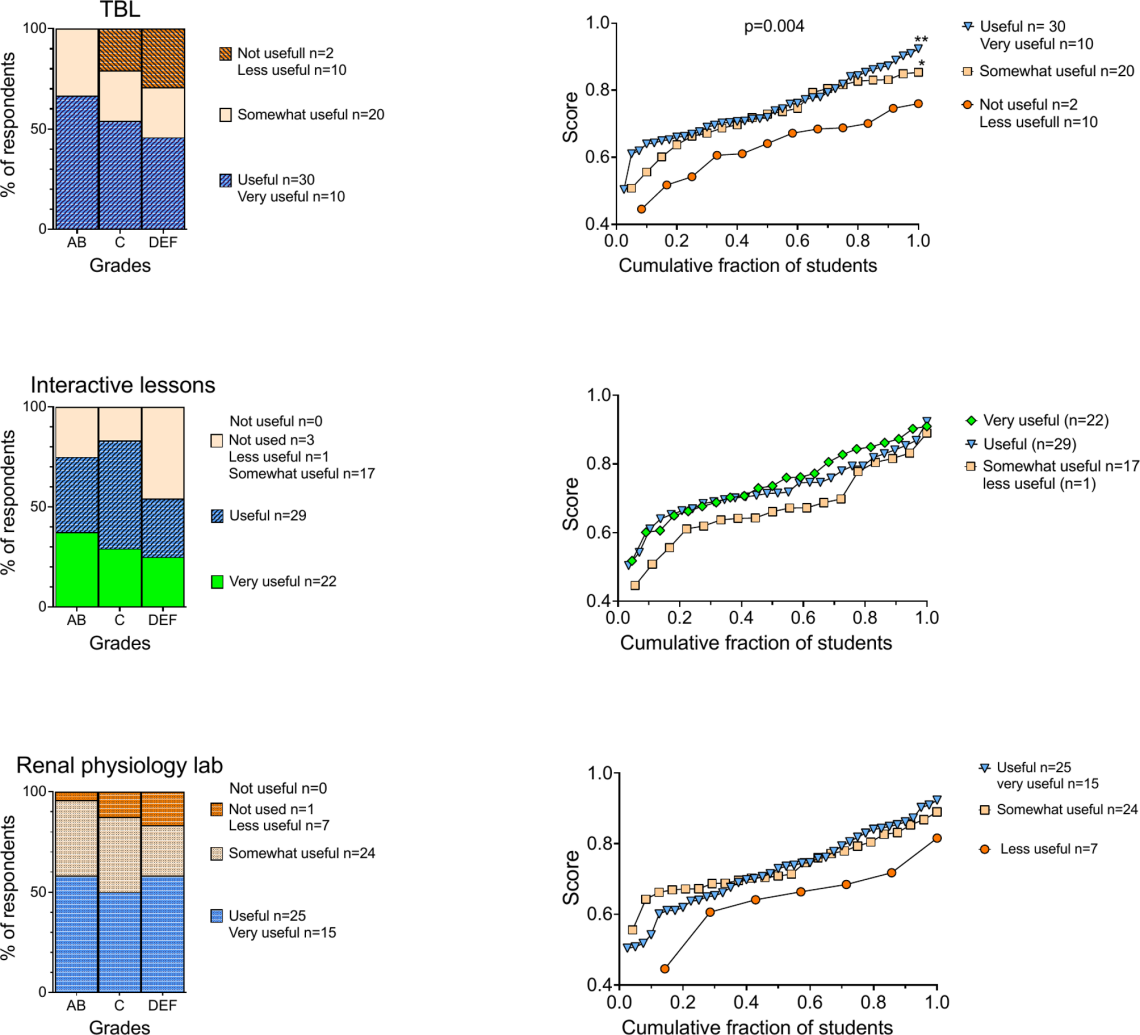
Comparison of the perceived usefulness of active learning methods and academic performance. The left panel shows the distribution of students’ preferences across different grade categories; high (AB), mid (C), and low (DEF) performers. The bars are divided into segments representing preference-cohorts as indicated by the legends. The right panels depict exam scores sorted from low to high as a function of the cohort-normalized number of students. **Upper panel**. Significant differences in exam scores were observed among the preference cohorts for ‘TBL’. Mean exam scores for the three cohorts (right panel): ‘Very useful/useful’; 74.6% ± 9.7%, n = 40), ‘Somewhat useful’; 72.8% ± 10.0%, n = 20, ‘Not very useful/not useful’; 63.4% ± 9.5%, n = 12. ANOVA: F (2, 69) = 6.121, p = 0.0036. Post hoc tests: **p = 0.0024 between the ‘Very useful/useful’ and ‘Not very useful/not useful’ cohorts, and *p = 0.0286 between the ‘Somewhat useful’ and ‘Not very useful/not useful’ cohorts. **Middle and lower panels.** No significant differences in exam scores were seen among the preference-cohorts for ‘Interactive lessons’ or ‘Renal physiology lab’

**Fig. 3:**
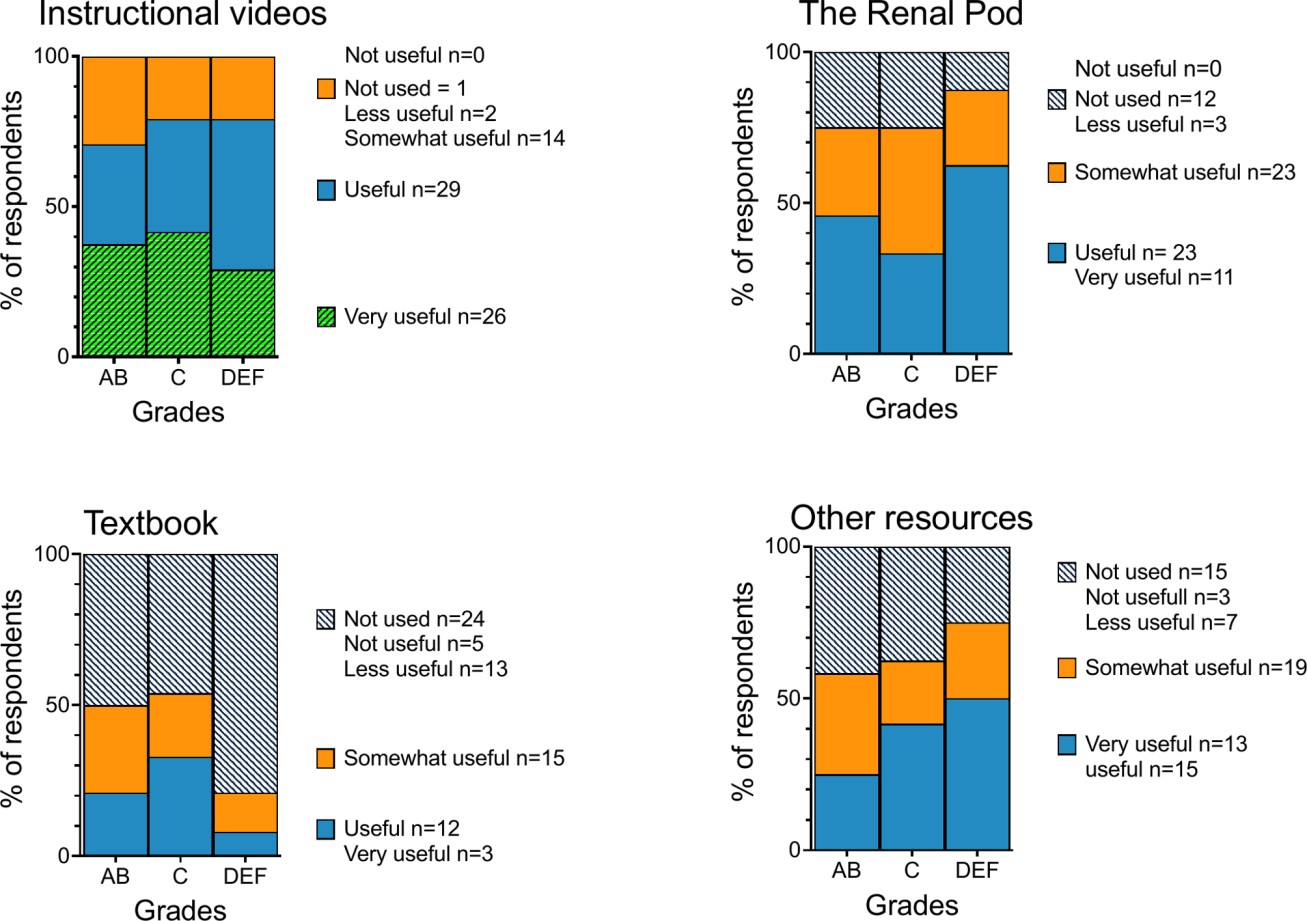
Comparison of perceived usefulness of indicated learning methods and academic performance (*p* > 0.05). See Fig. 2 for a detailed explanation of the panel structure

**Fig. 4:**
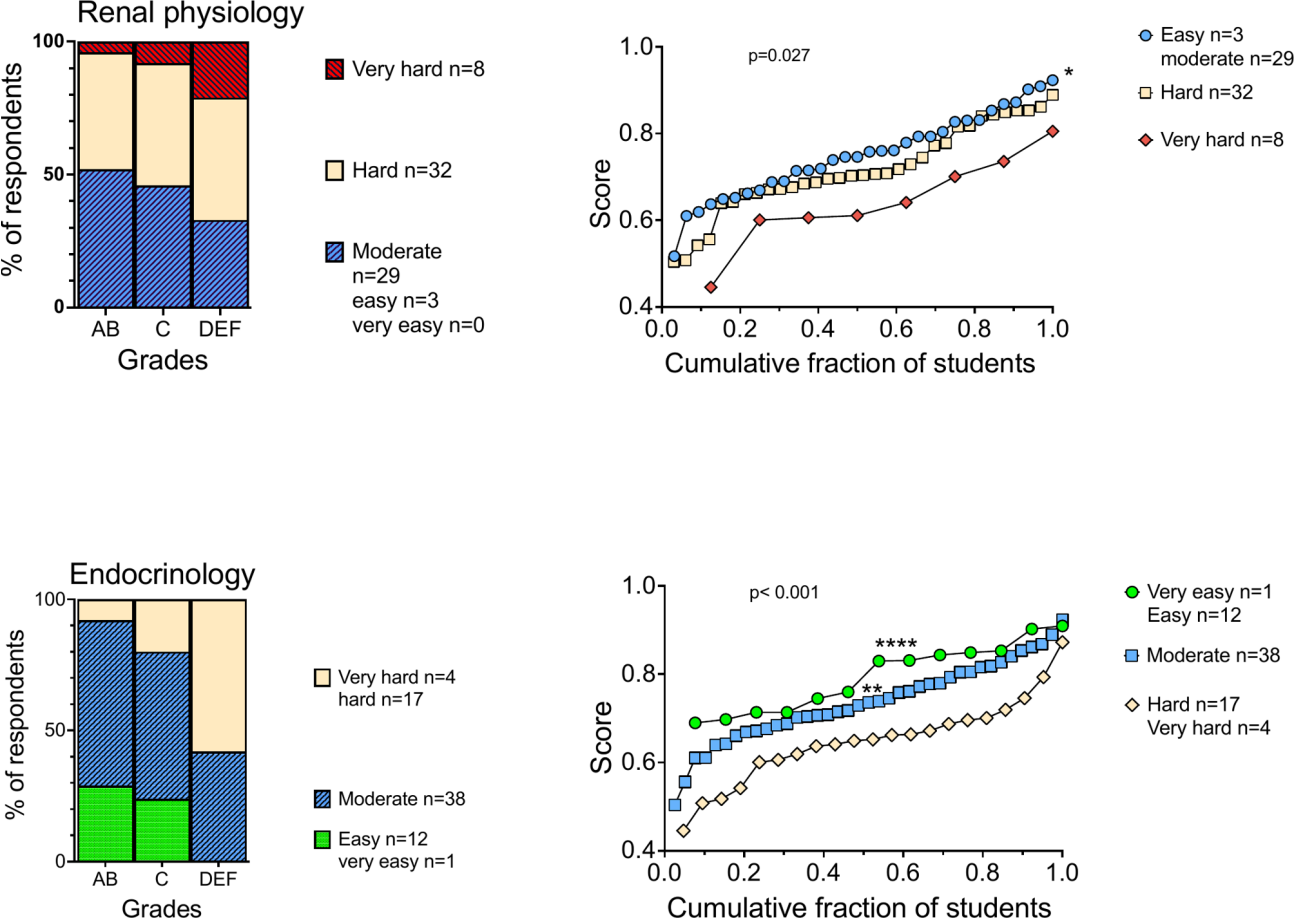
Comparison of perceived difficulty of renal physiology and academic performance. See Fig. 2 for a detailed explanation of the panel structure. **Upper panel**. Significant differences in exam scores were observed based on the perceived difficulty of renal physiology. Mean exam scores for the three cohorts (right panel): ‘Easy/moderate’; 75.1% ± 9.7%, n = 32, ‘Hard’; 71.8% ± 10.3%, n = 33, ‘Very hard’; 64.3% ± 10.8%, n = 8. ANOVA: F (2, 70) = 3.793, p = 0.0273. Post hoc tests: *p = 0.0228 between the ‘Easy/moderate’ and ‘Very hard’ cohorts. **Lower panel**: Significant differences in exam scores were observed based on the perceived difficulty of endocrinology. Mean exam scores for the three cohorts (right panel): ‘Very easy/easy’; 79.5% ± 7.8%, n = 13, ‘Moderate’; 73.8% ± 9.2%, n = 39, ‘Hard/very hard’; 64.9% ± 9.7%, n = 21. ANOVA: F (2, 70) = 11.49, p < 0.0001. Post hoc tests: ****p < 0.0001 between the ‘Very easy/easy’ and ‘Hard/very hard’ cohorts, and **p = 0.0017 between the ‘Moderate’ and ‘Hard/very hard’ cohorts

**Fig. 5:**
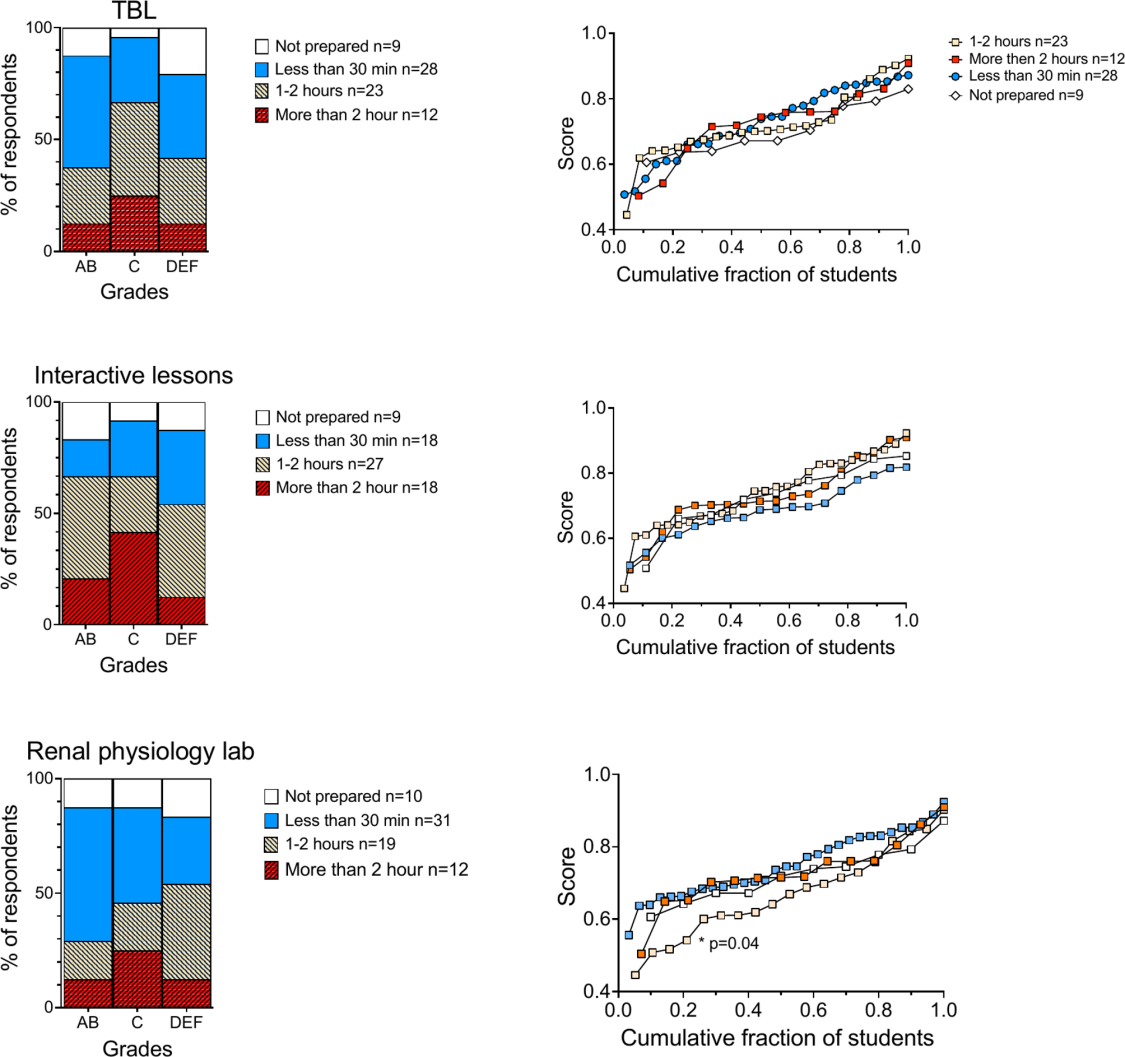
Comparison of time spent preparing for active learning sessions and academic performance. See Fig. 2 for a detailed explanation of the panel structure. **Upper** and **middle panel.** No overall significant difference for TBL and Interactive lessons (p > 0.05). **Lower panel.** Mean exam scores for the four cohorts (right panel) based on time spent preparing for the ‘Renal Physiology lab’: ‘Not prepared’; 72.4% ± 7.9%, n = 10, ‘Less than 30 min’; 75.1% ± 8.9%, n = 31, ‘More than 1 h’; 67.2% ± 12.5%, n = 19, ‘Anything more than 2hurs’; 73.0% ± 9.7%, n = 14. ANOVA: F (3, 70) = 2.498, p = 0.0667. Post hoc tests: *p = 0.0400 between the ‘Less than 30 min’ and ‘More than 1 h’ cohorts

**Fig. 6:**
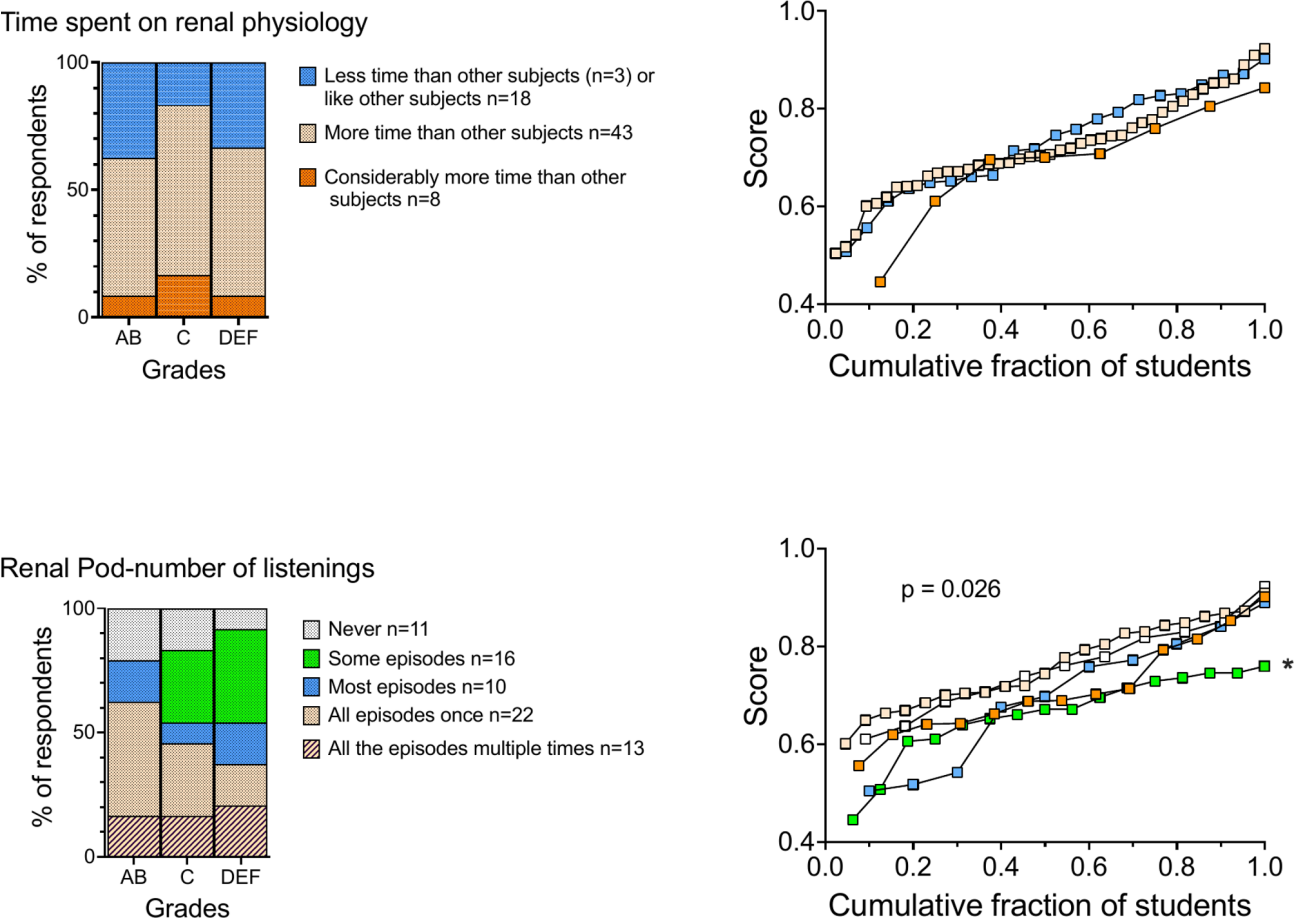
The impact of study time and podcast engagement on academic performance. See Fig. 2 for a detailed explanation of the panel structure. **Upper panel.** No significant difference in academic performance based on time spent studying renal physiology (*p* > 0.05). **Lower panel**. Significant differences in performance based on podcast usage. Mean cohort exam scores (right panel): ‘All the episodes multiple times’; 71.4% ± 10.0%, *n* = 13 ‘All episodes once’; 76.3% ± 8.7%, *n* = 22 compared to those who listened to only some episodes: 66.2% ± 8.7%, *n* = 16. ANOVA, F (4, 67) = 2.958, *p* = 0.0259) post hoc test: *p = 0.0217

**Fig. 7:**
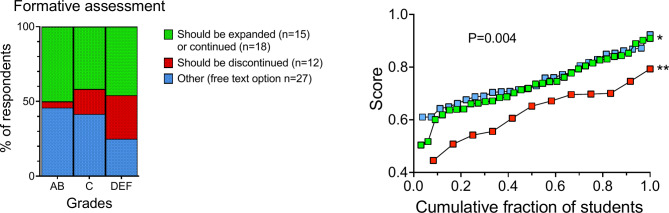
Comparison of academic performance and recommendations for the formative assignment, showing significant differences in exam scores based on students’ perceptions of the formative assignment. See Fig. 2 for a detailed explanation of the panel structure. Mean exam scores for the three cohorts (right panel): ‘Should be expanded or continued’; 73.2% ± 10.3%, *n* = 33, ‘Should be discontinued’; 63.4% ± 10.4%, *n* = 12, and ‘Other’; 75.0% ± 8.7% n = 27. ANOVA: F (2, 69) = 6.136, p = 0.0035. Post hoc tests: *p = 0.0117 between the ‘Should be expanded or continued’ and ‘Should be discontinued’; **p = 0.0029 between ‘Other ' and ‘Should be discontinued’ cohort

The survey responses were compared to academic performance, as indicated by exam grades and scores, to discern patterns in learning efficacy and resource utilization. Respondents were categorized into three performance groups based on their MED4 exam grade A-F: ‘high performers’ (A or B, *n* = 24), ‘mid performers’ (C, *n* = 24), and ‘low performers’ (D, E, or F, n = 24). For each group, the percentage of responses to each closed-ended question was calculated and presented as bar charts (Figs. 2–7). The respondents’ exam scores were then organized from lowest to highest and plotted to show the cumulative distribution across the response categories (right panels of Figs. 1–2 and 4–7). In cases where fewer than eight respondents selected the same option, exam scores from respondents in adjacent categories with similar responses were combined for a more robust data representation.

To further analyze the data, Likert-scale responses from the survey were converted into numerical values on scales of 1–3, 1–5, or 1–7, depending on the question. These numerical values were subsequently correlated with total exam scores and renal physiology scores using Spearman’s rho analysis. The ‘not used’ responses from Sect. 4 of the survey were excluded from this analysis. To address potential biases introduced by merging different response categories (e.g., ‘useful’ and ‘very useful’), we re-analyzed the data without merging the categories and observed similar patterns, supporting the consistency of our findings.

### Statistical tests

Exam scores are presented as the mean values ± standard deviations (SD). The number of participants (n) in the different response cohorts is indicated in the figure legends. The differences in exam scores between the whole class (*n* = 189), the sample population (*n* = 72) and the nonresponders (*n* = 117) were assessed using one-way ANOVA and linear regression analyses, assuming a normal distribution of scores. Differences in exam scores among the different response cohorts were evaluated using one-way ANOVA, with the assumption of homogeneity of variances being met, followed by Tukey’s post hoc test for multiple comparisons. Additionally, Spearman’s rho analysis was employed to examine the relationships between students’ preferences for various learning resources and their academic performance. We used Bonferroni correction for the multiple correlations to adjust for the increased risk of Type I error and report both uncorrected and adjusted p-values. All the statistical analyses were performed using GraphPad Prism software version 10.1.1. A p-value of less than 0.05 was considered to indicate statistical significance.

## Results

### Sample population and grade comparison

Out of 189 eligible MED4 students, 72 (38% response rate) completed the questionnaire and provided exam identifiers for anonymous grade linkage. The grade distribution centered around a ‘C’ for both the sample and the entire class, as shown in Fig. 1A. Regression analyses revealed a high degree of explanation (R² = 0.61) for the linear relationship between total exam scores and renal physiology scores across the whole class, the sample population, and the non-respondents. The slopes and intercepts were similar across these groups (*p* = 0.96), indicating a consistent pattern in the relationship between total scores and renal physiology scores. However, the sample population exhibited a tendency toward higher performance, particularly in the lower-performing group, as depicted in Fig. 1B. Compared to the nonrespondents (*n* = 117), the difference in total and renal scores was statistically significant (F (5, 750) = 10.24, *p* < 0.0001), indicating some degree of self-selection among the respondents.

### Perceived determinants of success in renal physiology learning

When evaluating factors that are critical to successful learning of renal physiology, 83% of the students across all the performance groups considered the communication skills of teachers to be ‘Very important’. Self-effort and teachers’ professional competence followed, with 67% and 58%, respectively, of the students rating these as ‘Very important’.

Notably, all the ‘high performers’ considered self-effort ‘Important’ or ‘Very important’, while 4% of ‘mid performers’ and 8% of the ‘low performers’ rated it merely ‘Somewhat important’. Teachers’ charisma was deemed the least influential, with 42% of ‘high performers’, 16% of ‘mid performers’, and 36% of ‘low performers’ reporting it ‘very important’.

Spearman’s rho analysis showed that students’ perceived importance of teachers’ professional competence positively correlated with their performance in renal physiology (rho, ρ = 0.23, *p* = 0.05), their perceived importance of self-effort (ρ = 0.25, *p* = 0.03), teacher communication skills (ρ = 0.23, *p* = 0.05), and the valuation of the following methods for learning renal physiology: TBL (ρ = 0.26, *p* = 0.03), interactive lessons (ρ = 0.29, *p* = 0.01), and instructive videos (ρ = 0.25, *p* = 0.04). However, these correlations were not significant after Bonferroni correction for repeated analyses (p adjusted > 0.05).

Additionally, self-effort was positively correlated with the perceived usefulness of interactive lessons (ρ = 0.32, *p* = 0.007, p adjusted > 0.05) and instructive videos (ρ = 0.30, *p* = 0.01, p adjusted > 0.05). In contrast, teachers’ charisma was negatively correlated to the time spent on preparing for the renal physiology lab (ρ = -0.34, *p* = 0.004, p adjusted > 0.05), and TBL (ρ = -0.24, *p* = 0.04, p adjusted > 0.05) as well as the valuation of ‘The renal Pod’ (ρ = -0.29, *p* = 0.01, p adjusted > 0.05) and ‘Renal physiology lab’ (ρ = -0.23, *p* = 0.05, p adjusted > 0.05).

Interestingly, the reported time ‘spent learning renal physiology compared to other subjects in MED4’ correlated positively to its perceived difficulty (ρ = 0.43, *p* < 0.001, adjusted *p* < 0.01) as well as difficulty of heart physiology (ρ = 0.45, *p* < 0.001, adjusted *p* < 0.01). Further, the perceived difficulty of renal physiology correlated positively with that of heart physiology (ρ = 0.51, *p* < 0.001, adjusted *p*  < 0.01) and negatively to the valuation of TBL (ρ = -0.34, *p* = 0.004, p adjusted > 0.05), textbook (ρ = -0.33, *p* = 0.02, p adjusted > 0.05) and recommendation for continuing formative assignments with feedback (ρ = -0.30, *p* = 0.04, p adjusted > 0.05).

### Linking academic performance with students’ valuation of learning tools

The most favored educational materials among the respondents were instructive video videos (96%), interactive lessons (94%), renal physiology labs (89%), and The Renal Pod (79%), each rated as ‘very useful’, ‘useful’ or ‘somewhat useful’.

As shown in Figs. 2 and 3, ‘high performers’ and ‘mid performers’ predominantly rated ‘Interactive lessons’ (79%), ‘Asynchronous videos’ (75%), and ‘TBL’ (60%) as ‘very useful’ or ‘useful’. Conversely, ‘low performers’ preferred ‘Asynchronous videos’ (79%), ‘The Renal Pod’ (63%), and ‘Renal physiology lab’ (58%) as their top learning resources.

Students who rated active learning methods (TBL, interactive lessons and the renal physiology lab) as most useful also obtained the highest exam scores (Fig. 2). This trend was particularly pronounced for TBL, where the average exam score in the ‘Very Useful’ and ‘Useful’ cohorts was 0.75 ± 0.10, compared to 0.63 ± 0.09 in the ‘Not Very Useful’ and ‘Not Useful’ cohorts (p = 0.002). The ‘Somewhat Useful’ cohort also outperformed those who found TBL ‘not useful’ or ‘not very useful’ (*p* = 0.029).

‘Other resources’, representing self-found online materials, and ‘Textbook’ were perceived as the least useful across all groups (Fig. 3). Notably, a trend emerged where ‘high performers’ seemed to value textbooks more than ‘low performers’ (21% vs. 8%), while ‘low performers’ showed a greater inclination towards ‘Other resources’ compared to ‘high performers’ (50% vs. 25%).

Spearman’s rho analysis further supported these observations, revealing positive correlations between overall MED4 exam score and students’ valuation of TBL (ρ = 0.28, *p* = 0.02, p adjusted > 0.05) and interactive lessons (ρ = 0.27, *p* = 0.03, p adjusted > 0.05). Conversely, MED4 exam score correlated negatively with perceived usefulness of self-found online resources (ρ = − 0.27, *p* = 0.04, adjusted > 0.05), suggesting that reliance on these resources might not be as beneficial for academic success.

### Perception of subject difficulty and its relationship with academic performance

The survey data showed that circulatory and renal physiology were the most challenging subjects, with only 4% of students finding them ‘Very easy’ or ‘easy’. Specifically, 71% rated circulatory physiology and 33% rated renal physiology as ‘difficult’ or ‘very difficult’. In contrast, nutrition was perceived as the least challenging, with 58% considering it ‘easy’ or ‘very easy’, and none finding it ‘very difficult’.

Students who perceived subjects as less challenging tended to perform better academically. For instance, only 4% of ‘high performers’ found renal physiology ‘very difficult,’ compared to 8% of ‘mid performers’ and 21% of ‘low performers’. This expected trend across disciplines is exemplified in Fig. 4, which shows that perceived difficulty in renal physiology and endocrinology is associated with lower exam scores.

These observations align with Spearman’s rho analysis, revealing negative correlations between MED4 exam score and perceived difficulty across the MED4 curriculum. Specifically, perceptions of difficulty in endocrinology (ρ = − 0.52, *p* < 0.0001, p adjusted < 0.01), heart physiology (ρ = − 0.35, *p* = 0.002, p adjusted > 0.05), renal physiology (ρ = − 0.30, *p* = 0.01, p adjusted > 0.05), and digestive system (ρ = − 0.25, *p* = 0.04, p adjusted > 0.05).

Similarly, endocrinology showed a pattern where perceived difficulty correlated positively with perceived difficulty in other topics such as digestive system (ρ = 0.46, *p* < 0.001, p adjusted < 0.01), respiratory system (ρ = 0.29, *p* = 0.01, p adjusted > 0.05), Heart (ρ = 0.27, *p* = 0.02 p adjusted > 0.05 and circulation (ρ = 0.26, *p* = 0.03 p adjusted > 0.05. These correlations, along with negative correlations with MED4 exam score (ρ = − 0.52, *p* < 0.001, p adjusted < 0.01) as well as renal score (ρ = − 0.34, *p* = 0.003, p adjusted > 0.05) and the valuation of TBL (ρ = − 0.24, *p* = 0.04, p adjusted > 0.05) support the conclusion that students’ perceptions of subject difficulty are aligned with both their preferred learning methods and their academic outcomes in the broader MED4 curriculum.

### The influence of preparation time on academic performance in active learning settings

The left panels of Fig. 5 indicate that mid-performers (grade C) are more likely to dedicate extensive time to preparation (more than 2 h), whereas high performers (grades A and B) tend to be more efficient with shorter preparation times (less than 30 min), Among low performers (grades D, E, and F), the most frequently reported preparation time is less than 30 min, followed by 1–2 h, with a notable portion also reporting no preparation.

As shown in Fig. 5, (lower right panel), an 11% lower exam score was noted in the cohort spending 1–2 h (0.672 ± 0.125) compared to those spending less than 30 min (0.751 ± 0.089) on preparation for ‘The renal physiology lab’ (adjusted *p* = 0.04). This suggests that while time spent preparing is a factor, the quality and effectiveness of study strategies are likely more crucial for academic performance.

Notably, a positive correlation was observed between the reported time students spent on interactive lectures and their perceived usefulness (ρ = 0.38, *p* = 0.01, p adjusted > 0.05), as well as with the time dedicated to preparation for these lectures (ρ = 0.51, *p* < 0.0001, p adjusted < 0.01) and the ‘Renal physiology lab’ (ρ = 0.43, *p* < 0.0001, p adjusted < 0.01). Conversely, a negative correlation was found with ‘self-found online resources’ (ρ = -0.38, *p* = 0.008, p adjusted > 0.05). However, as with time spent on other available learning methods, no correlation was found between reported preparation time and exam score.

These findings highlight the interconnectedness between the time spent on preparation for active learning methods, their perceived value, and academic performance.

### Comparison of the time spent on renal physiology relative to other subjects

While approximately 70% of respondents reported spending more time on renal physiology compared to other MED4 subjects, this increased study time did not translate into significantly different academic performance across the cohorts (Fig. 6 upper panel). Specifically, the average exam score for respondents who spent considerably more time on renal physiology was 69.6% ± 12.4%, compared to 72.2% ± 9.9% for those who spent more time, and 73.4% ± 11.0% for those who spent a similar amount or less time studying the subject (*p* = 0.69).

### Podcast engagement and its impact on academic performance

As depicted in Fig. 6 (lower left panel), a substantial majority (85%) of respondents engaged with ‘The Renal Pod’, with 63% having listened to most or all episodes at least once, and 18% reporting multiple listenings of all episodes. Notably, high-performing students (AB) in contrast to their low-performing peers (DEF), predominantly listened to all the episodes once (46%) or not at all (21%) suggesting a strategic approach to podcast utilization.

The results revealed a statistically significant differences (*p* = 0.026) in academic performance based on podcast usage (Fig. 6, lower right panel). Specifically, students who listened to all episodes once achieved higher mean exam scores (76.3% ± 8.7%) compared to those who listened to only some episodes (66.2 ± 8.7%). This finding suggests that moderate and consistent engagement with the podcast is more beneficial for academic success than sporadic listening.

Despite these nuanced usage patterns, a strong desire for more podcast-based learning resources was expressed across all performance levels, with 69 out of 72 respondents advocating for broader podcast availability.

### The impact of formative assignment perceptions on academic performance

The introduction of the formative assignment pilot in Spring 2022 allowed us to examine its impact on the academic performance of MED4 students. This assignment involved collaborative problem-solving and individual feedback sessions, as described in the study context.

Students across all grade categories expressed a preference for the expansion or continuation of the formative assignment. Specifically, 50% of AB, 42% of C, and 46% of DEF students supported this view (Fig. 7, left panel). However, 29% of DEF students recommended discontinuation compared to only 4% of AB students (red segments in Fig. 7, left panel). Additionally, students could respond with ‘other’, which required a free-text explanation. The majority of these free-text responses also supported the formative assignment, indicating a general preference for its continuation or expansion.

The right panel of Fig. 7 shows that students advocating for the continuation or expansion of the formative assignment had a 13% higher mean exam score (73.2% ± 10.3%) compared to those who preferred its discontinuation (63.4% ± 10.4%, adjusted p = 0.01). ‘Other’ respondents, who largely supported the pilot, had a 15% higher mean exam score (75.0% ± 8.7%, adjusted *p* = 0.003). Altogether, these results indicate potential benefits of the formative assignment approach.

## Discussion

Our study explored the correlations between time spent studying, students’ perceptions of the utility of learning resources, and their academic performance within the MED4 curriculum, aiming to identify characteristics of successful learners in medical education. While our focus was on renal physiology, primarily due to its comprehensive use of active learning resources, the exam results we analyzed encompassed the entire MED4 curriculum, including scores in specific subjects.

The findings reveal that there is a good correlation between students’ performance in renal physiology and their overall academic success and an interconnectedness between time spent on preparation for active learning methods, their perceived value, and academic performance. This suggests that the learning strategies and resources employed in this challenging subject have transferable value to other academic areas.

In line with our findings, Bin Abdulrahman et al. [17] highlighted that highly effective medical students tend to employ structured study habits and strategic use of learning resources, which are critical for academic success. In their study, top-performing students reported regular revision, active engagement with learning materials, and a preference for diverse study methods. These habits align with our observation that high-performing students (grades A and B) are more likely to engage with active learning resources such as Team-Based Learning (TBL), interactive lessons, and formative assignments.

We observed that both high and low performers prepared for teaching, with a tendency for most students to report investing more time in learning renal physiology compared to other subjects. However, a significant correlation between time spent studying and academic performance was not evident. This observation, reflecting the heterogeneity within both high and low performer groups, highlights how individual differences in learning strategies, cognitive capabilities, and motivation levels can influence the effectiveness of study time. Although we did not measure students’ prior knowledge, it seems that the essence of what distinguishes more effective learners is not the quantity, but rather the quality of their study time. Aligning with this concept, findings from West and Sadoski emphasize the importance of effective management skills in higher education [18]. Several other studies support this [19–22]. For example, Liles et al reported that 77% of students achieving ‘A’ grades reviewed lecture material on the same day, compared to just 25% of those with ‘C’ grades. Additionally, high achievers were more likely to attend classes, limit online lecture usage, and study for 6–8 hours daily [21]. In addition to time management skills, West & Sadoski suggest that self-testing skills may improve academic performance [18]. This aligns with the extensive body of work on retrieval practice, where Roediger and Butler, Karpicke and Blunt, and Dobson have demonstrated the efficacy of retrieval practice in enhancing long-term retention and academic performance [23–25]. This may partly explain why TBL was one of the most popular resources among high performers in our study, as it provided questions that enabled students to test their understanding of the subject. Emke et al. also found that Team-Based Learning can enhance short-term knowledge acquisition, though its long-term effects are less clear without continued practice [26].

Bansal et al. also found that high-performing students had a preference for deep and strategic learning strategies, contrary to low-performing students, who mostly used the surface approach to learning [19]. The classification of deep and surface learning approaches, along with their implications for educational practice, is based on the seminal work of Marton and Säljö [27]. While the deep approach emphasizes understanding concepts and relating ideas, the surface approach emphasizes route memorization [28].

While TBL shares some similarities with flipped classrooms, such as pre-class preparation and active learning during class, there are key differences. In a flipped classroom, students typically engage with lecture material at home through videos or readings, and then participate in interactive activities during class to deepen their understanding. TBL, on the other hand, emphasizes team-based activities where students work together to solve problems and apply concepts during class. Both approaches aim to enhance student engagement and learning outcomes, but TBL specifically focuses on collaborative learning and peer-to-peer teaching, which can foster a deeper understanding through group discussion and problem-solving.

We observed an 11% improvement in academic performance equivalent to an absolute difference of 20 points out of 179, among respondents who favored TBL and who supported the continuation of formative assignments compared to those who found TBL less favorable and recommended discontinuation of the formative assignment pilot. Notably, formative assignments were not conducted in renal physiology but in other subjects such as cardiology, endocrinology, and digestion and nutrition. Therefore, the potential influence of formative feedback on renal physiology exam performance was not directly evaluated. This suggests that some students might be less adept at interpreting and constructively utilizing the feedback inherent in TBL and formative assignments. In the literature, these abilities are described as feedback literacy and denotes “the understandings, capacities and dispositions needed to make sense of information and use it to enhance work or learning strategies” [29]. Furthermore, our findings align with studies on flipped classroom methodologies in physiology, which found improved performance in new students [30] and enhanced students’ learning effectiveness and skills like self-study and problem-solving [31].

In addition, low performers tended to rank ‘self-effort’ and ‘Textbook’ as a less important factor for learning renal physiology and to rely more on ‘self-found online materials’ and ‘The Renal Pod’ compared to their better performing counterparts. Their perception of subject difficulty, especially in challenging areas like renal physiology, was negatively correlated with academic performance. This attitude, combined with their lower satisfaction with TBL and formative assignments, characterizes low performing students and aligns with their preference for passive learning resources [20, 32, 33]. This preference may stem from their perception of active learning methods as more cognitively demanding, as found by Deslauriers et al. [7].

While these methods are associated with better academic performance, as supported by the present study, the perceived challenge might drive some students towards passive resources which they believe are less demanding. This highlights a potential disconnect between student perceptions of learning resource effectiveness and their actual impact on academic performance. Moreover, the distinction aligns with the findings of Roediger and Karpicke, who demonstrated that students’ predictions of their own learning are often uncorrelated with actual performance, emphasizing the critical role of retrieval practice in consolidating learning [34].

An interesting finding in our study is the distinct podcast listening habits among the different student performance groups. While low performers rated ‘The Renal Pod’ as highly useful and 92% engaged with it, their listening patterns varied, with a notable proportion not completing the episodes. In contrast, high performers predominantly listened to all episodes once or not at all, suggesting that a strategic approach to using podcasts as a learning tool is associated with better academic performance in medical education.

Previous literature shows conflicting reports on whether educational podcasts can help students improve their examination scores [35]. McCarthy et al. argue that podcasts have potential as a supplement to existing curricula, where they can fulfill the need for interested learners [36]. However, if students perceive podcasts as a *replacement* for other learning resources, they risk reduced learning efficiency. In particular, since students often engage in other activities while listening to podcasts and listen at double speed, the educational impact may be limited [36].

In our study, textbooks were perceived as the least useful learning resource, regardless of academic performance. Previous research has indicated that replacing textbooks with evidence-based articles and summary questions does not have a negative impact on students’ academic performance or satisfaction [37]. Furthermore, studies have revealed that older students do not advise new students to buy many textbooks but rather focus on PowerPoints from professors, old exams and summary notes [38]. Since teachers typically create exams, this may lead to bias toward the lecture material and undermine students’ motivation to use textbooks. Moreover, when experiencing curriculum overload, many medical students may be compelled to adopt coping strategies and surface approaches to learning.

Although this study was conducted at a single medical school, the findings may have broader applicability. The curriculum at the University of Bergen shares similarities with many European medical schools, particularly in the preclinical phase, where integrated curricula and active learning methods are widely used. This suggests that our findings could provide valuable insights for other educational contexts employing similar strategies. Extending this research to a variety of settings would further validate these results and enhance their generalizability.

Our findings suggest that educators in similar contexts consider placing greater emphasis on active learning resources, such as TBL and formative assignments, to foster deeper learning. By strategically integrating active teaching methods into the curriculum, medical schools may enhance student engagement and academic performance, particularly in complex subjects like renal physiology.

In summary, our findings highlight the relationship between student engagement with learning materials and academic performance. Although we measured correlations between reported use or preferences and academic success, rather than the quality of engagement, we argue that the strategic use of active learning methods and resources like ‘The Renal Pod’ is more critical than the quantity of study time. This emphasize the need to teach students effective study habits. Consequently, educational approaches should extend beyond content delivery to include fostering skills in resource selection, time management, and feedback utilization. Finally, our study implies that assignment methods should be carefully considered for their educational impact, as they are likely to influence student learning behavior and outcomes.

### Limitations

This study, while providing valuable insights into the learning resource preferences of successful learners in medical education, has several limitations.

#### Correlation vs. causation

It is important to acknowledge that correlation does not imply causation. While our correlation analyses highlight relationships between learning resource usage and exam scores, these associations may be influenced by other factors such as students’ intrinsic motivation, prior knowledge, and engagement levels.

#### Self-selection bias

The response rate of 38% raises concerns about potential self-selection bias, as the respondents might have been more engaged or motivated, potentially influencing the study’s outcomes. This is evidenced by our finding that the sample population exhibited a higher average total score compared to non-respondents, indicating some level of self-selection among the respondents. Even though the sample population’s academic performance in renal physiology was consistent with the overall class, the self-selection bias might limit the generalizability of our findings. Future studies with larger sample sizes are needed to validate these findings.

#### Exam scores vs. deep understanding

High exam scores do not necessarily equate to a deep understanding of the subject matter. The exam’s broad scope and its combination of essay questions and reasoning-based MCQs aim to reduce the likelihood of achieving good results solely through memorization. Further studies with practical assessments and long-term retention tests are recommended to better evaluate deep understanding and learning achievements.

#### Survey bias

The reliance on surveys for data collection introduces potential biases as students might not accurately report their study behaviors. These biases should be considered when interpreting the correlations between learning resource preferences and academic performance.

#### Demographic data

The absence of demographic data leaves potential factors influencing students’ preferences and performances unexplored. This decision was made to maintain participant anonymity and streamline the survey process, but we recognize that demographic factors could influence the results. The absence of questions regarding traditional lectures, which were not used in renal physiology teaching, may also limit the comprehensiveness of our findings.

#### Generalizability

While the study was conducted in a single institution, the relevance of the findings has been discussed in detail earlier. However, caution should still be exercised when applying these results to different educational contexts without further validation.

## Conclusion

Students who perform well on exams tend to prefer active learning strategies and make strategic use of resources, suggesting that the quality of study time impacts academic performance more than the quantity. Based on these findings, we recommend that educators consider integrating student-active teaching methods into the curriculum and providing guidance on effective study practices to enhance learning outcomes.

## Electronic supplementary material

Below is the link to the electronic supplementary material.

Supplementary Material 1

## Data Availability

All data supporting the findings of this study are contained within the manuscript, the accompanying figures, and the supplementary file. Additional data related to this study are available from the corresponding author upon reasonable request.

## Abbreviations

UiB: The University of Bergen
ECTS: European Credit Transfer and Accumulation System
MCQs: Multiple choice questions
SD: Standard deviation
TBL: Team-Based Learning
CBL: Case-Based Learning
