# Supplementary material for: Study preferences and exam outcomes in medical education: insights from renal physiology
Source: BMC Med Educ. 2024 Sep 6;24:973. doi: 10.1186/s12909-024-05964-4 (PMC11380206; doi:10.1186/s12909-024-05964-4)
Supplement: Supplementary file 1 — Supplementary Material 1 [file 12909_2024_5964_MOESM1_ESM.pdf]

Supplementary information

Survey MED4 2022

Renal physiology is a demanding subject in MED4 because it intervenes in so many of the body's systems and contains many new terms. Through this questionnaire, we want to investigate how medical students learn renal physiology and what are the preferred teaching tools.

The survey is anonymous, but we ask for candidate numbers to find possible connections between answers and exam results. This can provide useful information about the effect of the individual learning resources. We do not have a list of names and there will be no link between name and candidate number. The candidate number will be deleted from the data set and will not be published.

It is important to specify that the responses are processed completely anonymously and cannot be linked to individuals. The answers are to be used exclusively for research purposes to improve teaching. Answering the survey is voluntary, and you can withdraw your consent at any time.

We greatly appreciate your participation in the survey. Your answers can help shape the future of teaching at the University of Bergen. If podcasts are a useful learning tool, there will be podcasts in several subject areas under the auspices of UiB.

Candidate number:

_____

1. **How important are the following factors for you to learn renal physiology?**

	Not important	Less important	Somewhat important	Important	Very important
Your self-effort	❑	❑	❑	❑	❑
Teacher's professional competence	❑	❑	❑	❑	❑
Teacher's charisma	❑	❑	❑	❑	❑
Teacher's communication skills	❑	❑	❑	❑	❑

2. How easy/difficult do you think these topics in physiology have been?

	Very easy	Easy	Moderate	Difficult	Very difficult
Renal	❑	❑	❑	❑	❑
Heart	❑	❑	❑	❑	❑
Circulatory system	❑	❑	❑	❑	❑
Digestive system	❑	❑	❑	❑	❑
Respiratory system	❑	❑	❑	❑	❑
Endocrinology	❑	❑	❑	❑	❑
Nutrition	❑	❑	❑	❑	❑

3. How much time have you spent learning renal physiology compared to other subjects in MED4?

- ❍ Significantly less time than other subjects
- ❍ Less time than other subjects
- ❍ Equal to other subjects
- ❍ More time than other subjects
- ❍ Significantly more time than other subjects

4. **How useful do you find the following learning resources for learning renal physiology?**

	Not used	Not useful	Less useful	Somewhat useful	Useful	Very useful
TBL	❍	❍	❍	❍	❍	❍
The Renal Pod	❍	❍	❍	❍	❍	❍
Renal physiology lab	❍	❍	❍	❍	❍	❍
Textbook	❍	❍	❍	❍	❍	❍
Asynchronous videos	❍	❍	❍	❍	❍	❍
Interactive lessons (with assignments, kahoot etc.)	❍	❍	❍	❍	❍	❍
Online resources I have found myself	❍	❍	❍	❍	❍	❍

5. Which statement best describe The Renal Pod episode length?

❍ Not used
❍ Too short
❍ Fine
❍ Too long
❍ Comments.. ______

6. How often have you listened to The Renal Pod?

❍ Never
❍ Some episodes
❍ Most of the episodes
❍ All episodes once
❍ All episodes several times

7. If you find podcast to be a valuable learning resource, why is that?

- ❑ Time-efficient
- ❑ A good supplement to other learning resources
- ❑ Can listen multiple times
- ❑ Short episodes that cover the most important aspects
- ❑ Other... (write in the text box) _____

8. If you don't find podcast to be a valuable learning resource, why is that?

- ❑ Not time-efficient
- ❑ Redundant learning resource
- ❑ Lacks the visual aspect
- ❑ Too short episodes
- ❑ Too long episodes
- ❑ Other... (write in the text box) _____

9. How much time have you spent on average preparing for these different teaching methods in renal physiology?

	Have not prepared myself	Less than 30 minutes	More than one hour	More than two hours	More than three hours	More than four hours	More than a school day
TBL	❍	❍	❍	❍	❍	❍	❍
Renal physiology lab	❍	❍	❍	❍	❍	❍	❍
Interactive lessons (with assignments, kahoot etc.)	❍	❍	❍	❍	❍	❍	❍
Asynchronous videos	❍	❍	❍	❍	❍	❍	❍

10. Would you like podcasts in other subjects at medical school as well?

❍ Yes ❍ No

11. Formative assessment has been a pilot this semester. What do you think about this teaching?

- ❍ Should be discontinued
- ❍ Should continue
- ❍ Should be expanded to more topics
- ❍ Other... (type in the text box)_____

12. What was favorable about the teaching in MED4?

(Text box) _____

13. How can teaching in MED4 be improved?

(Text box) _____
